# Part III. Analecta

**Published:** 1827-04

**Authors:** 


### PRACTICAL MEDICINE;

BEING

### The Spirit of the Medical Journals,

*Foreign and Domestic;*

WITH COMMENTARIES.

### PART III.
### ANALECTA.

"Nihil est aliud magnum quam multa minuta."

### 1. DIVISION OF MEDICINE INTO PHYSIC AND SURGERY.

Mr. Lawrence's introductory lecture to his Spring Course has been published in the Lancet, No. 181, and the lecturer has exerted all his talents and rhetoric to prove, that no real distinction or line of demarcation can be drawn between physic and surgery, whether we look to education or practice. We shall here bring forward a document, shewing how far Mr. Lawrence has been original in his arguments and illustrations, on this point of medical jurisprudence. The following paper was written and published by the Editor of this Journal *ten years ago*, and may be seen in the 4th volume of the Medico-Chirurgical Journal, pages 342—7. It will be evident that Mr. Lawrence has taken the arguments, the illustrations, and almost the identical expressions of this paper for his introductory lecture.

"The boundaries between physic and surgery have not till this moment been defined—a strong presumption that nature does not sanction them! The distinction of internal and external diseases is any thing but correct or satisfactory. Gout, for instance, alternates with erysipelas. In the first form the physician claims it as his own; in the second, the surgeon steps in, and demands the treatment. But is not the erysipelas in this case, and indeed in almost every other case, dependent upon an internal, or what is termed a constitutional cause? and can it be, with any degree of safety, treated as a topical affection? Here, then, must not Physic and Surgery be united?*

"The same may be said of anthrax, and a great majority of inflammatory affections, whose prominent and apparent seats are indeed external, but

* "When we look to the nature, causes, and treatment of diseases, the distinction between internal and external appears pre-eminently absurd. Internal causes produce external diseases, and external agencies affect internal parts. Erysipelas, gout, carbuncle, œdema, exemplify the former—pneumonia, catarrh, and many forms of rheumatism, the latter."—*Lawrence.*

whose cause and origin are internal. If the surgeon treats them without the aid of physic, he does wrong: the physician considers them as out of his province; and if he does undertake the cure, he must use surgical means with medicinal. How preposterous would it be to have a physician combating the cause, and a surgeon the effect—or, more correctly speaking, to employ the physician to lop off one arm of the disease, while the surgeon is busy in extirpating the other? Either, then, the physician and surgeon must be of one opinion, and consequently be equally informed on the subject, or the practitioner must unite physic and surgery in his own person. In short, the distinction between internal and external diseases has no more foundation in nature or truth, than that between diseases of the *right and of the left side* of the body.*

"The allotment of all diseases requiring *manual* assistance to the department of surgery would create equal confusion. A patient is seized with violent pneumonia. The physician is sent for: he declares that venesection is immediately necessary, and the paramount remedial measure. But in these times the surgeon no longer obeys the orders of the physician; they are independent of each other, and act only from their own judgment. But, granting that a bleeder is procured to act under the physician, and things go on well for a time, it may and does happen that pneumonia terminates in empyema. Then comes the difficulty. It is surely as necessary (indeed it is much more so) to know *when*, as how, to perform an operation. The surgeon, on whom the responsibility of the operation rests, must select *his* time for the performance, and for that purpose *ought* to know the nature and exact state of the case as well as the physician. Here physic and surgery must be again united.

"On the other hand, let us take the purest surgical case—a compound fracture of the leg. When the surgeon has reduced the fracture, and comfortably adjusted the limb on a pillow, his work is far from being finished. The local will soon produce general derangement, and the whole fabric of the constitution will be in danger without physical aid. Here, then, as a contrast to the first example, the physician will be set to work on the *effect*, while the surgeon is occupied with the *cause!* What matchless absurdity. But will it be said, that the febrile commotion excited by the fracture differs in any essential point, either in its nature or treatment, from that occasioned by an internal inflammation, as of the lungs? By no means. Yet the latter is placed exclusively under the physician, and the former under the surgeon. The distinction, then, between local and general diseases, cannot be maintained, since organs the most distant are sympathetically influenced, and constantly drawn into consentaneous derangement. All diseases, in fact, are local in their origin. They commence in one organ or set of organs, spreading to all others with more or less rapidity, according to the degree of lesion and importance of function in the organ first affected.

"A late eminent French writer (Richerand), who by the bye seems no great friend to the separation of physic from surgery, proposes a new classification of diseases for the more accurately distinguishing the limits of the two branches. All diseases, according to him, may be said to consist in physical derangements, organic affections, and lesions of the vital powers —'lesions des propriétés vitales.' To the former only, or *physical derangements*, consisting of wounds, dislocations, obstructions, &c. and

---

* "If you must have a distinction, separate the disease of the *right and left side*, or of the upper and lower half."—*Lawrence.*

their consequent operations, he would confine the province of surgery, leaving a wide field, indeed, to Messieurs les Medecins. " Les lesions physiques sont presque toujours le resultat de l'action d'un corps exterieur sur le notre ; ce sont des effets méchaniques d'une cause qui l'est elle-même. Les plaies ou solutions de continuité produites par un instrument tranchant, piquant ou contondant ; les fractures occasionées par l'alongement forcé du tissu osseux ; les luxations amenées par la violence des mouvemens imprimés a nos parties ; une hernie, resultat de l'effort méchanique des puissances expiratoires, reconnaissent une cause physique, apporte a nos fonctions un obstacle mechanique, et reclament pour leur guérison, l'emploi des moyens du meme ordre.'

" According to this scale, the surgeon might still, as he does now, claim the treatment of a chancre, as being the effect of '*un corps exterieur* ;' but, as soon as the virus had shifted its seat from the prepuce to the tonsil, the physician would say, ' Now is my turn !' But does the treatment of a venereal sore in the former situation differ from that in the latter ? And if not, why not ? seeing that they are treated by different orders of the profession ? Here physic and surgery are evidently interlocked. Well may Monsieur Richerand say, ' Ces matières *Medico-Chirurgicales* peuvent, ce me semble, être comparées à ce crepuscule qui conduit du jour à l'obscurité.' But, taking M. Richerand on his own ground, I would ask him, can the external impression of rain, and a piercing cold wind on the surface of the body, checking the perspiration, breaking the balance of the circulation, and inducing pneumonia, be considered strictly, and philosophically, less '*une action d'un corps exterieur sur le notre,*' than the puncture of a bayonet, producing the same effect ? Yet, in one case, the inflammation ' le *resultat* de l'action, &c.' is medical, and in the other it is surgical ! What are intermittent fevers—typhus, the plague itself, but the effects of foreign bodies on our own ? What is hydrophobia ? What is traumatic tetanus, but '*le resultat de l'action, &c.*'

" In no possible way can we find any natural or just distinctions between physic and surgery ; and yet I do not object to the distinctions in *practice.* It is against the idea of a difference in *education and study* that the above remarks are levelled. It is morally impossible that a man can be a good physician without knowing the principles of surgery ; and it is still more absurd, to think that a man can be a good surgeon without being intimately acquainted with the principles of physic.* If it be objected that an intimate acquaintance with the minutiæ of both branches is too much for one person to acquire, I answer, 1st. in the words of Richerand, ' l'etendue de la science ne justifie point les *limites arbitraires* que l'on voulu tracer entre ses diverses parties.' 2d. I am of opinion, that it is much more easy to acquire a profound knowledge of both branches, than of either one separately : and for the reasons already stated, that the two sciences are interlocked at all points, even the apparently most distant ; for instance, *pneumonia and fracture.* Hence, to attempt to learn the one without the other, is to tie up one of our arms when we commence a mechanical trade !

" Is it not absurd to say, that a *moderate* knowledge of anatomy and surgery is useful to the physician, and deny that a *minute* knowledge would be still more so ? As for the surgeon, he cannot move a step in safety, without an intimate knowledge of all the laws of the animal œconomy, which the physician studies and observes.† His very operations for the removal

---

* " These remarks apply only to the *study* of the medical profession, which must be learnt as a whole."—*Lawrence.*

† " I cannot quit this subject, without impressing on you most earnestly of diseases that would otherwise prove mortal, are inflictions that often require the most consummate knowledge of physic to counteract. What madness, then, for the surgical student to direct his attention so exclusively, as he too often does, to the study of anatomy and surgery, when he is assured by grey hairs, that nine-tenths of his future professional labours (whatever line he may take) will be what are termed purely medical! Let him compare M. Richerand's list of 'Physical Lesions' with the long black catalogue of organic and vital; and he will see that it is but a drop of water in the ocean.

"Not that anatomy and surgery are not to be most minutely studied; but I would say, that these branches are only parts of a grand whole, *all* of which must be studied with *equal care*, or every branch is imperfect.

"Let us glance at the great disorders of the three principal cavities of the body. Is idiopathic phrenitis more dangerous and difficult to treat than that from a fracture of the skull? Are idiopathic convulsions more terrible than those from a splinter of bone driven through the coverings of the encephalon? Is idiopathic tetanus more fatal than traumatic? In what consists the difference between idiopathic and traumatic pleuritis, pulmonitis, carditis, diaphragmitis, hepatitis, gastritis, splenitis, enteritis, peritonitis, cystitis?—None. Or, if there be any difference, the difficulty and the danger are on the side of surgery.

"What then remains? Fever? Does the hectic of the lungs exhibit a greater spectacle of horror and emaciation than the hectic of lumbar abscess, or disease of the hip-joint? Is the inflammatory fever resulting from aërial vicissitudes to be compared with that supervening on a lacerated member or crashed knee-joint, where the dire commotion threatens every instant to annihilate the living fabric at a blow? The typhoid fevers differ in no respect from the ulterior stages of inflammatory and symptomatic fevers.

"Seeing, then, that the greatest and most dangerous diseases, both in physic and surgery, to which humanity is subject, are completely identified, is it not evident that the surgeon, in acquiring a knowledge of *these*, must, in course, acquire a knowledge of the minor and intermediate maladies, which are reckoned *Medico-Chirurgical* by both professions? And, is it not evident from these premises, that the surgeon, if properly qualified, must add to the *science* of the physician the *art* of operating? Anatomy, physiology, and surgery, are merely the first steps by which the student gains a footing on the hill of science. When he can unravel the brain without a light, or take up the external iliac blindfold, he is scarcely advanced a day's march on the great journey of medical science. These, which are termed, and justly, elementary branches, are little more than the alphabet of medicine, though they are considered at the beginning as the ultima thulé of professional knowledge. The surgical student thinks himself armed at all points; but every year's subsequent experience shews him more and more his own ignorance!

"In fine, physic and surgery are only parts of a grand whole; one cannot be properly known without the other, no more than navigation can be learnt without the aid of arithmetic. The distinctions between the two branches cannot be drawn or maintained, even in the largest cities. To be convinced of this, let any one sit down by the side of Cooper and Abernethy for a day, and he will see these illustrious surgeons lopping off nine surgical

---

the advantages—nay, the necessity, of studying *medicine* comprehensively."
—*Lawrence.*

cases with the quicksilver pill and infusion of cascarilla, for every one which they remove by the knife or by caustic. But what is more extraordinary, he will see patients of all ranks afflicted with purely *physical* diseases, resort to surgeons for advice. Hence it is evident, that the general sense of mankind acknowledges not the artificial divisions which we have made in the profession.

"Nevertheless, the separation of physic from *operative* surgery, whatever violence it may do to Nature, is probably productive of good to both branches. There are few minds capable of resisting the seductions of the scalpel, where a bold and surgical genius is bestowed by nature. The eclat of operations eclipses the slow, and dull, and too often unsatisfactory march of medical science; and constant application to one line begets, of course, a facility and dexterity that cannot be otherwise attained. The deficit in other branches is not felt in a great city or public institution, where the patient is surrounded by all ranks and degrees of the Faculty. On the other hand, the physician, liberated from the anxieties of the operation-room, brings a calm unruffled mind to the investigation of the infinitely varied phænomena of disease. A close attention to these, generates, without a doubt, a quickness of apprehension, a fertility of resource, and an accuracy of diagnosis, to which but the *favoured few* of surgeons can pretend. But, except the purely *operative* part of surgery, medical and surgical *practice* is completely blended, for the best of all reasons, that medical and surgical diseases are essentially the same. Hence it is incumbent on the young physician to *study* surgery at least, and to dissect as assiduously as though he were designed for the operation room; while the surgical student may be assured that *medicine* is his sheet anchor at last; and that whatever degree of accuracy he may attain as an anatomist, or dexterity as a surgeon, yet, without pathology and therapeutics, these brilliant acquisitions will avail him little in his professional journey through life."

We leave it to the public, and to Mr. Lawrence himself, to decide whether all the arguments and illustrations brought forward in this celebrated introductory lecture, have not been anticipated, and even more forcibly urged, in the article which we have here republished. Mr. Lawrence's lecture is characterized in the Lancet as a "brilliant effort," to shew how impossible and foolish is the arbitrary distinction attempted to be drawn between physic and surgery. But we shall soon see that the same arguments, coming from another quarter, will be considered, by the same impartial judge, as ridiculous and imbecile. The learned lecturer has not only trodden the same ground as the writer of the article here republished, but has come to the identical conclusions on all the different points—namely, first, that the distinction between physic and surgery is baseless: secondly, that the education should be the same for both, or as nearly so as possible: and, lastly, that the various branches being first studied, the division of labour may afterwards be advantageous to the public, and beneficial to science.*

* "I do not contend that every one should practise all parts; and I am fully aware that the field is far too extensive for one individual to cultivate the whole minutely, with a view to further improvement. No doubt that one, who has received a general medical education, may improve a particular department, if he should have his time fully occupied with it; and that circumstances of taste, convenience, situation, and public opinion, may thus lead to subdivisions in practice with advantage to the public and benefit to science."—*Lawrence.*

### 2. ILEUS, FROM CALCULOUS CONCRETIONS.

[From the Spanish.]

A lady of Barcelona, 50 years of age, of very sedentary habits, and subject to hysteria, was seized, on the 8th March, 1822, with acute pain in the epigastric and right hypochondriac region, under the false ribs. The pain was so violent that she was forced to cry out repeatedly, and could not bear the slightest pressure on the parts. There was no tension or swelling of the parts. The tongue was moist and clean—no fever—pulse feeble—no motion for 24 hours previously. *Lavements—Hoffman's anodyne liquor.* The pain continued unabated during the 9th, notwithstanding various means were used. On the 10th, the pain was less acute, but there were nausea, and even vomiting, on taking some aliment. No stools from the enemata. 11*th.* Much the same; vomiting continues, accompanied by hiccup—abdomen distended—no passage in the bowels—some fever. 12*th.* Stercoraceous matters were thrown up—no stool—belly more distended. 13*th.* Four ounces of quicksilver were swallowed—and tobacco-smoke was injected *per anum.* The vomiting and other symptoms continued. In the evening, stools and urine passed freely—the vomiting ceased—the belly became soft, and the pain nearly disappeared—good sleep during the night. 14*th.* The vomiting returned. 15*th.* Bilious stools came away, and the other symptoms were mitigated. The patient had some tranquil sleep, from which she awoke free from fever, pain, or thirst, and with some appetite. 16*th.* The abdominal pains returned, and again disappeared, after a copious evacuation of fæcal matters. But, instead of the quicksilver being rendered by stool, the patient passed a calculus, the size of a large chesnut, having a smooth and polished surface. 18*th.* The patient evacuated another calculus, similar to the former, the size of a pigeon's egg. Next day, a third was passed, of triangular figure, and as large as the second one. In washing this last it broke to pieces, and disclosed a nucleus, around which the concentric layers had formed. The patient was now in full convalescence. On the 20th, the mercury was evacuated, along with much fæcal matter. She was soon quite well. These concretions were analyzed by Professor Ballcells, of Barcelona, and 30 grains gave 20 of cholesterine—5 of inspissated bile—4 of a resinous substance, and one of loss.—*Archives.*

The Spanish author and M. Julia-Fontanelle, who translates the case, seem to consider these calculi as intestinal concretions. We have not the smallest doubt that they were biliary. It is highly probable that they had passed from the gall-bladder into one of the small intestines—or, perhaps, into the colon, by ulceration. Such an event has happened in this country, as was proved by dissection afterwards—the patient having died of another disease.

---

### 3. SYNCOPE ANGINOSA.

In one of the sittings of the Academy of Medicine, M. Chantourelle read the case of a French nobleman, which deserves some commemoration, although the precise nature of the disease was not verified, as it usually is in France, by dissection.

*Case.* M. le Duc ———, had enjoyed good health till the age of 58 years. In the month of March, 1826, he began to experience some inconvenience when he walked fast, and on the 12th of the same month he fell down in a state of syncope, which lasted nine or ten minutes, and then he revived, but the action of the heart, and the pulse at the wrist, continued imperceptible for some time, the breathing being very short. By baths, sinapisms, leeches to the anus, and cordials, the circulation was restored, in some degree, by the third day. On the fifth day he was bled from the arm, when the pulse became still more developed, and the action of the heart could be felt all over the left side of the chest. During the following days the patient was able to get up, take some light food, and even go out in his carriage. At this time, some moral emotion caused a great tumult in the circulating system, and he was again bled. He complained that he could not sleep—the intellectual faculties were quite free. On the 24th of March, the Duke went to church, and in kneeling down, fell into another fit of syncope, from which he never recovered.—*Archives, Dec.*

M. Chantourelle saw the corpse four hours after death. The face was pale, but along the whole left side of the thorax there was an enormous sanguineous infiltration. The body was not allowed to be opened. M. Chantourelle informs us that the disease with which the Duke was afflicted, was considered successively to be aneurism of the heart, apoplexy, angina pectoris. We were rather surprized to find that some members of the Academy maintained that the Duke died of pulmonary apoplexy. There cannot be a doubt that the disease was dilatation of the heart, most probably of the passive kind. Rupture of the organ, we should suppose, took place, and gave origin to the appearances of bloody infiltration in the chest.

### 4. TRANSFUSION IN UTERINE HÆMORRHAGE.

Mr. Brown, a very able practitioner in the Borough, has communicated an interesting case of this kind, in the eighth Number (New Series) of the Medical and Physical Journal. The particulars are as follow :—A female, 30 years of age, was delivered of her tenth child at a quarter before one o'clock, P.M. of the 31st of December. She had always suffered from uterine hæmorrhage on these occasions, and an alarming flooding soon succeeded the present delivery. Mr. B. introduced his hand, and the uterus soon contracted and stopped the hæmorrhage; but the mischief was produced. An alarming collapse ensued, which required brandy, æther, and camphor. These gave only temporary relief. Convulsions and vomiting took place, and again the collapse threatened the life of the patient. Dr. Blundell, Mr. Doubleday, and Mr. Waller were summoned. The latter only could be found. He arrived at half past two o'clock, an hour and three quarters *post partum*. The following was the state of the case :—Lies on her back, with a death-like expression of countenance—extremities of marble coldness—little animal heat in the chest or abdomen—laborious and stertorous respiration—eyelids closed, and eyes insensible to light—pupils fully dilated—jaw fallen—pulse imperceptible at the wrist, and even in the carotids. It was Mr. Brown's firm conviction that the woman was dying; but it was properly determined to give her the chance of transfusion. Thirteen drachms of blood were injected, in the usual manner, at twenty-five minutes to three o'clock. No particular effect seemed to be produced by this operation. In five minutes another injection, of thirteen drachms, was effected; and now the action of the radial artery was felt—the breathing became more free—but the pupils remained dilated, and the eye evinced no sensibility to light. In ten minutes more a third injection was made, the aggregate quantity amounting to about five ounces of blood. The improvement was now more evident. She moved her extremities and uttered a faint ejaculation. Deglutition (before extinct) now enabled her to take three tea-spoonfuls of brandy. It was not, however, till after the fourth injection, that the amelioration was most conspicuous. The pupil now readily contracted on the admission of light—the chest was fully expanded at each inspiration—the pulse was 120, and equable. She seemed in pain about the left hypogastric region. She recognized her husband and others—and, in short, the urgent danger was evidently over. We need not pursue the details of the case. As is usual after such extreme states of collapse, a pretty violent re-action succeeded, and required even detractions of blood from the head; but the patient completely recovered.

We think that every unbiassed person must here recognize, not merely the safety, but the utility of transfusion. There are some, however, who cannot be convinced. If the patient recovers after transfusion, it was owing to the efforts of Nature, or the effects of other means :—If she dies, the inutility of transfusion is, of course, manifest. There is no species of evidence which can carry conviction to the minds of these people, and, therefore, we shall not waste our own time, or the time of our readers, in argumentation. We advocated the propriety of transfusion, ever since we became acquainted with the first attempts which were made, and we have no cause to withdraw or change our opinions on this subject. Much praise is due to the gentlemen engaged in this transaction, which we consider as one of the most unequivocal proofs of the safety and efficacy of transfusion, which have yet come before the public.

### 5. RETENTION OF URINE; FALSE PASSAGE.

There is a case of this kind reported lately from Guy's Hospital, and, if the report be correct, there is a lesson contained therein, which surgeons would do well to bear in mind. We know that some surgeons of high repute have great confidence in the powers of nature, when retention of urine is to be treated. A man may be two or three days unable to make water, or, at all events, unable to discharge it as fast as it is secreted—he may be suffering great pain—the bladder may be felt above the pubes—there may be an impossibility of introducing a catheter,—and yet the surgeon will say —"Oh! let him alone, the water will dribble through." It will often do so, we are aware; but it will sometimes dribble through the urethra, behind the stricture, into the cellular membrane—or inflammation and suppuration may take place in the neighbourhood of the urinary passage, and when the matter makes its way into the urethra, urine will make its way into the cavity of the abscess, and the consequences may be fatal. The following case, which we shall greatly abridge, appears to have been an instance of this kind.

*Case*. A middle-aged man was admitted into Guy's, on November 15, 1826, having laboured for some days, under partial retention of urine, and passed none during the preceding 18 hours. Catheterism was attempted (by Mr. B. Cooper) in vain. The patient was bled in a warm bath till faintness was induced; but still the catheter could not be passed into the bladder. Some urine dribbled through, however, after the bath. Still the bladder was felt distended above the pubes, where much tenderness is evinced on pressure. The pulse is small, tongue furred—hiccup. Calomel and opium at bed-time. More attempts were unsuccessfully made with the catheter on the 16th. Leeches to the pubic region. In the evening, Mr. Callaway (who happened to be going round) succeeded in introducing the catheter, and drew off four pints of high-coloured urine. Much relief was now, of course, experienced, and the catheter was mostly kept in the bladder. On the 20th, there were two shivering fits; on the 26th, there was great irritability and restlessness, with irritative fever, and much pain about the membranous part of the urethra, to which leeches were applied.

*27th.* Pus was discharged with the urine. Irritative fever continues. *28th.* A fluctuating swelling, the size of a walnut, was perceived, immediately anterior to the scrotum, which might be made to disappear by pressure. Swelling and tenderness in the perineum. *29th.* Swelling in front of the scrotum increased—urine constantly dribbling away. On the 4th Dec. we find that "the abscess has greatly enlarged—it is harder to the touch—and the integuments, to some extent around, have a dark red colour; whilst, in the centre of the swelling, there is a gangrenous spot, nearly the size of a shilling." The constitutional symptoms were in unison with the local affection. An opening was made, and some ill-conditioned pus, mixed with urine, came away. We need not pursue the details any farther. The event may be easily anticipated. The patient died on the evening of the 7th December.

*On dissection*, a stricture, of considerable firmness, was found in the bulbous portion of the urethra. An abscess had formed externally to the urethra, partly anterior, partly posterior to the seat of stricture. "There was a false passage in the urethra, large enough to admit of the point of a catheter, and it was of about two inches in length; this false passage commenced anteriorly to the stricture, and terminated at a short distance behind it, so that, in all probability, the catheter had taken this course behind, or rather beneath the natural channel, and had thus, as it were, evaded the stricture." The nates were found to be in a sloughy state to some depth; but not apparently from infiltration of urine.—LANCET, No. 179.

This is a very remarkable case; at least in one point of view. It is not often that the point of the catheter, having once ruptured the urethra, and got into the cellular substance, finds its way back into the urethra, beyond the obstruction. We know, indeed, that a stricture may be bored through, as it were; but this curvetting of the catheter out and in of the passage is a phenomenon to which we never heard of any thing, "*simile aut secundum*," before. It is abundantly evident that this new or circumambulant passage, if it really existed as such, must lie at the door of Mr. Callaway, who succeeded in reaching the bladder after Mr. Cooper had failed. Mr. Callaway will doubtless give the public some information on this curious point.

At all events, we think it will be allowed that the danger of puncturing the bladder above the pubes—or of taking the chance of the water "dribbling through," without any assistance, was much less than that of using such force with a catheter as was sufficient to rupture the urethra. This last accident should not happen. The choice will then be between paracentesis vesicæ and the dribbling process. For our own parts, we should much prefer the knife, since there is scarcely any danger in puncturing the bladder above the pubes, but great danger in temporising, as the foregoing case sufficiently shews. It is more than hinted by the reporter, that the swelling under the urethra was not opened in due time. We should have been glad to know what other morbid phenomena presented themselves among the vital viscera. The local disease was inadequate to destroy life, without other sympathetic lesions in one or more of the great organs of the body.

---

### 6. SCIATICA CURED BY TURPENTINE.

The ol. terebinth. is one of the *heroic* medicines which our Continental neighbours have ventured to import from this land of specifics. Venesection is evidently performed with a bolder hand since their intercourse with the English—and even calomel is kept in their boutiques, if not actually prescribed, on some rare occasions.

In the November RÉVUE MEDICALE, M. Piorry has stated a case of severe femoro-popliteal neuralgia, cured by the medicine at the head of this article.

Madam A——, aged 40 years, had been harrassed with violent pains, of a lancinating character, shooting from the pelvis along the thigh, in the direction of the sciatic nerve, and down even to the foot, ever since the month of February, 1826. The pains were increased by the slightest movement, and they were unaccompanied by any visible change in the colour, size, or temperature of the parts. The respiratory and digestive functions were healthy; but an evening access of fever obtained, and terminated in a profuse perspiration in the night. The pains were exasperated during the evening accession. This series of symptoms continued for six months, and resisted the various means that were employed by her physicians. She came under M. Piorry's care on the 6th August. He applied forty leeches in the line of the sciatic nerve—the part was enveloped in a large cataplasm—and baths were afterwards administered. Diluent drinks and low diet were enjoined. A slight amelioration ensued, and the leeches were twice repeated. Still the amendment was very inconsiderable, and, indeed, the third application of the leeches exasperated, rather than soothed the pains. The extract of lettuce was then employed, in full doses, but with only temporary benefit. On the 1st September a large blister was applied, which caused the pain to cease entirely for a time, again to be renewed with equal violence. The oil of turpentine, in the dose of one drachm thrice a day, was given, suspended in syrup and water, by means of yolk of egg. The medicine produced heat at the epigastrium, but no vomiting the first day. The second day it vomited the patient, and the dose was given only twice a day. The patient could feel that the turpentine remained long on the stomach, and it took away the appetite. On the third day the neuralgia was mitigated, and in four days it was quite dissipated, there only remaining a sense of formication, which also disappeared in a day or two more. The patient thought herself cured—began to walk about her room—and left off the medicine. The pains returned, though in a slight degree, and the turpentine was resumed for a few days more, when the recovery was complete.

It appears, from M. Piorry's account, that this medicine is becoming very popular in Paris. If its taste could be disguised, it would prove a valuable addition to our list of therapeutical agents. In our own practice, the patients have complained more of the terebinthinate eructations than of the mere deglutition of the medicine.

We are not informed whether the carbonate of iron was employed in the above case, prior to M. Piorry's attendance. We suspect that it would have cured the disease as speedily as the turpentine, and with much less inconvenience.

We were recently in attendance on a young gentleman who had long been afflicted with violent accessions of palpitation, pain in the region of the heart, in the upper part of the sternum, in the throat, and down along the left arm, as far as the elbow. He had read several medical books, and was convinced that he had aneurism of the heart or some of the large vessels. He, therefore, nearly starved himself, and certainly was a most deplorable looking object, being afraid of walking across his room, lest the aneurism should burst. By this system, and by the purgation and other depletive measures which he pursued, the disease was evidently exasperated. It usually came on in the afternoon, and would sometimes last the whole night, during which the action of the heart could be heard by the bye-standers. When we first saw him the heart was nearly quiet. The chest sounded well in all directions—the action of the heart was heard over only a very moderate space—and no unusual noise accompanied the ventricular or auricular contractions. As he had had no previous illness of any consequence—as he never had rheumatism—and as he had experienced some severe moral afflictions, we were convinced that the disease was of a nervous character.

His stomach was very much out of order, in consequence of the vegetable and fruit diet to which he confined himself, from apprehension of aneurism. The diet was changed—and tonics were given. The symptoms were mitigated, but the paroxysms of palpitation continued to return daily. We then ordered him a drachm of carbonate of iron in some compound confection of senna, (which, by the way, is a very convenient vehicle) twice a day. In three or four days the paroxysms became trifling in degree. The dose was increased to three drachms daily, and he was soon well.

### 7. AXILLARY ANEURISM.

Mr. Liston has detailed some cases of Aneurism in our elder brother of the North, the Edinburgh Journal for January, from which we shall notice one, where the subclavian artery was tied, but unsuccessfully.

*Case.* J. M. J. æt. 43, after travelling from 150 miles in the Highlands, applied, Sept. 12th, 1826, with an aneurismal tumour, the size of a football, in the right axilla. It passed upwards beneath the clavicle, raising it considerably, and downwards to below the border of the axilla, pressing in the ribs and flattening the chest. Limb of the natural temperature, but paralysed: its upper part hard—the lower œdematous, as was the side of the chest. It appears that about Christmas, 1825, he had fallen on the ice with his arm stretched out; that this was followed by violent pains, which were thought to be rheumatic, and that it was only ten weeks previously that he was made aware of the nature of his complaint. On the 14th, Mr. Liston proceeded to tie the subclavian, assisted by Mr. Mackenzie, and in the presence of Dr. Monro, Dr. Sanders, Dr. Malcolm, and other gentlemen. On exposing the artery, it was found to be much enlarged, and on withdrawing the aneurismal needle, a gush of blood followed, which was restrained by pressure with the finger, and soon stopped. The ligature was tightened, and pulsation in the tumour ceased. Apprehending, however, a return of the hæmorrhage, Mr. L. tied the vessel again three-fourths of an inch nearer the heart, and close to the edge of the scalenus anticus. Here the arterial coats were found, if possible, still more diseased.

All went on well for some days, but on the fifth evening the pulse rose from 90 to 120, with increase of pain in the tumour and arm. V. S. ad ℥viij.—anodyne. On the morning of the thirteenth day, some oozing from the wound; in the evening, oozing again, with relief to the pain and throbbing at the root of the neck, and diminution of the tumour. Next day, oozing of putrid blood again, and the patient sank.

*Sectio Cadaveris.* The tumour was diminished nearly one half in size, but still was of enormous extent. The vessel had given way just below the origin of the thoracica humeraria, and from that point to the edge of the scalenus, it was firmly attached to the tumour, and, in fact, contributed to the formation of its coats. The wound was filled with coagula, and the upper ligature was found detached, embracing a slough, and lying loose among them. The under ligature was still attached, though ulceration had occurred here also, to a considerable extent. This ligature lay in the sac behind the subclavian, and from this perforation had proceeded the hæmorrhage, which occurred on withdrawing the needle until the opening in the sac had in all probability been plugged up by coagulum. At neither of the points of deligation was there much appearance of attempt at reparation. There was no clot in the artery, except at the upper opening, which was soft and bloody; and though the vessel was a good deal contracted, the adhesions were slight; the axillary vein also necessarily passed over the inner part of the sac, adhering firmly to it, and obliterated about three inches below the clavicle. The tumour extended, by a process two inches in diameter, down to the bend of the arm in the substance of the biceps. The brachial artery below was filled by a firm coagulum; the anastomosing vessels much enlarged; the aorta and innominata were double their original size, and their coats thick and pulpy. Adhesions between the pleuræ on both sides.

*Remarks.* The fatal event was evidently owing to the morbid state of the artery; and from several cases of axillary aneurism which we have seen, where the coats of the artery were, as here, much diseased, we fear that operations on the subclavian can never be attended with general success. Indeed, looking to the anatomical and *pathological* difficulties which harass the operator, our only wonder is, that he has been so successful as he has. Mr. Liston doubts the accuracy of Mr. Wardrop's operation on the carotid. He says, "I should think it would puzzle Mr. W., and those who put faith in the efficacy of deligation beyond the sac, to say what effect would have been produced on this aneurism, by including the contracted and obstructed humeral artery in a ligature." But the parallel is hardly a fair one. In Mr. W.'s case, the disease was in its commencement—in this it was far advanced. From the carotid no branches are given off, there is nothing to keep up the current—in the axillary artery it is the reverse. The *principle* of Mr. W.'s operation is to check the circulation through the sac, but tying an obstructed humeral artery could not do this, for the anastomosing vessels were all enlarged; and to bring the case quite into the same circumstances as the carotid, it would be necessary to tie them. Again, the objection to the new operation, of its inefficacy in this case, would apply equally to the common operation, for it really *was* inefficacious.

To the paper of Mr. Liston a plate is appended, which shews the morbid appearances clearly and satisfactorily; and, from the details of the case, we are sure, that if dexterity on the part of the operator could have saved the patient, this would not have been recorded amongst the fatal instances of axillary aneurism.

### 8. CLINICAL MEDICINE.*

The subject of medical education has lately excited, and deservedly so, great attention in this country. The clinical education of the student is one of the most important portions of medical education, because on it will greatly depend the future success and reputation of the practitioner. Dr. Clark, whom we have often introduced to the notice of our readers, is well calculated to offer suggestions for the improvement of clinical instruction in this country, in consequence of his long residence in Italy, and his travels on the continent, with the professed view of gleaning useful information on this interesting subject. We hope the patrons and professors of the Edinburgh University will give an attentive perusal and consideration to the suggestions here thrown out, and that they will not allow themselves to be "compelled to *follow* in the march of improvement, when they should have *led*." If they permit private interest and cabal to drown the voice of honour and renown, that famous university may live "to see her halls deserted in the department of science to which she has been indebted for her

* Observations on the System of Teaching Clinical Medicine in the University of Edinburgh, with Suggestions for its Improvement, &c. By James Clark, M.D. 8vo. pp. 30, *sewed*. 1827.

most extended celebrity." She may, indeed, exultingly point to the increased, or increasing number of her medical students; but numerical calculations are dangerous documents to build upon in such cases. Circumstances may render it convenient or necessary to frequent a market for some time after its goods have been deteriorated in value; but other markets may ultimately spring up to rival the old.

Dr. Clark has confined himself, in this pamphlet, to the subject of *Clinical* Instruction, and begins by portraying some of the best plans pursued on the continent, where the greatest attention is paid to this important item of medical education.

Two methods of teaching clinical medicine are adopted in the continental schools—the Hospital Clinic—and the Poly-clinic, or Ambulatory Clinic. The latter corresponds exactly with our dispensaries. In both these institutions, however, the system differs in some very material points from that in use at Edinburgh, or indeed in London. The chief difference consists, 1st, in the more advanced pupils having a part in the treatment of the patients; 2dly, in the greater length of time required of pupils to attend clinical medicine; 3dly, in the manner of appointing the clinical professors.

In the Hospital Clinic, the elder pupils are entrusted to attend to certain patients, of whom they may be said to have the charge, under the immediate inspection of the professor. These pupils draw up the histories of the cases, and are examined on the nature of the disease by the professor, in the presence of the other pupils. "They are also required to point out the symptoms which more especially characterize the disease, and serve to distinguish it from others which it most nearly resembles, and with which it might be confounded; and, finally, to give the prognosis and method of treatment. Those pupils continue to attend the same patients; and at every succeeding visit they examine, under the eye of the professor, the state of the symptoms, inquire into the effects of the remedies prescribed, &c. Of the whole case they keep a faithful record. If the disease should prove fatal, the attending pupil is further required to state, previously to the examination of the body (a thing which is never omitted), the morbid conditions which he expects to find, and what he considers to have been the cause of death." 6.

There can be but one opinion as to the great advantage which the pupil must derive from such a discipline; but we fear the temper of John Bull, who subscribes to, and becomes a governor of, an English or Scotch hospital, will not permit such delegation of treatment to the pupil, except on emergencies, when the physician or surgeon is out of the way. We have little hope, therefore, that this clinical regulation will be introduced into our English hospitals, at least for the present.

The *Poly-clinic*, or dispensary practice, is very general over Germany; and at some of the universities, it is the only source of clinical instruction. Those patients who can attend the Dispensary are prescribed for there; acute diseases are attended at the patient's houses.

"The pupil is first exercised in examining the patients that come to the Clinic, under the observation of the professor, and is required to state the nature of the disease, its treatment, &c., as in the Hospital Clinic. The treatment being agreed upon, the pupil writes the prescription, which is examined, modified if necessary, and signed by the professor. After a time, the pupil is intrusted with the care of the out-patients. He is required to draw up an accurate history of each disease under his care, which is submitted to the inspection of the professor, as are also the reports of the progress and treatment of the case. Moreover, when he finds himself in difficulty, or the case appears to the professor to require it, the clinical assistant accompanies him to see the patient, and assists him with his advice. In urgent cases, I believe, the professor also visits the patient; and where the disease proves fatal, he superintends the examination of the body, a practice to which, on the continent, objections are very rarely made." 8.

We are acquainted with some officers of public dispensaries, in this metropolis, who closely approximate to the foregoing plan, and who consequently are of more real utility to the pupil, than any officer of a public hospital can possibly be under existing regulations.

"Let us now compare the Edinburgh Clinic with those described. The patients in the wards are here visited once a day by the professor and pupils. The cases are carefully drawn up by the physician's clerks, and the pupils are permitted to copy them into their own case books. The successive daily reports may be added at each visit, as they are dictated at the bedside of the patient by the professor. To see and hear the professor examine and prescribe for the patients is all that the pupil, generally speaking, has to do with them*; and it is not always an easy matter for him to attain this limited and second-hand communication; the crowd being frequently so great as to deprive a considerable proportion of the pupils of the benefit of seeing and hearing distinctly, either the professor or his patient. There are two clinical lectures given each week, in which the professor explains the nature of the cases, the treatment, &c. These lectures form an excellent part of the clinical system of Edinburgh, and are too much neglected in the foreign universities in general. They are, however, too few in number. A patient may be received into the clinical ward, and pass through the whole course of an acute disease, before the professor has an opportunity of making any remarks on the case in these lectures; but even though made more frequent than they are, they never can, in my opinion, entirely compensate for the want of remarks on the cases, in the clinical wards, such as are made in most foreign clinics. In Edinburgh, the number of patients to be examined, in the period of time to which the visit is limited, renders it impossible to make any remarks of consequence." 11.

Abroad, the pupil is generally required to attend to clinical medicine for four sessions, or two scholastic years—at Edinburgh, only six months' attendance on the clinical course is necessary, and the same length of time at any respectable hospital. Abroad, the graduate must pass a considerable time, after obtaining his degree, before he can enter on the practice of his profession—that time being either in an hospital, or with some practical physician. After the completion of the period of probation, he again undergoes an examination, as to the progress he has made in practical medicine, before he can be admitted to the *Libera Praxis* of his profession. We are ashamed to compare, or rather to contrast, this with what we see done in this country.

We have only one more point to touch upon in this short article. In foreign universities, the clinical professor receives his appointment "expressly in consequence of his qualification to teach *practical medicine*." His duties, as clinical professor, are regarded as by far the most important, and in the exercise of this branch he is regularly occupied during the whole academical year. In Edinburgh, the clinical professors hold other chairs—

---

* "It is true the pupils may, I believe, visit the patients in the clinical wards in the intervals of the visits; but were many to avail themselves of this privilege, the patients labouring under acute diseases would not benefit by the practice."

are changed every three months—and cannot devote nearly enough of time to the clinicum. But this is not the worst of the case. The number of patients under each physician renders it absolutely impossible that he can properly examine and explain their cases to the pupils. There ought to be double or treble the number of medical officers, in consequence of which there would be fewer wards for each, and the swarm of pupils would thus be divided into smaller bands. They would then have some chance of seeing and hearing what was going forward.

The same observations apply, *à fortiori*, to the London hospitals, where the rush of pupils, with the surgeons at least, renders access to clinical instruction almost nugatory. The various defects above-mentioned, are proposed by Dr. Clark to be remedied in the following manner:—

"1st, By increasing the period during which the candidate for a medical degree shall be required to attend to clinical medicine (as without sufficient time he cannot possibly acquire a knowledge of practical medicine, however fair the opportunities held out to him may be); 2ndly, By increasing the number of clinical professors in office at the same time; 3dly, By diminishing the number of patients under the charge of each professor; 4thly, By increasing the number of clinical lectures; 5thly, By making the office of clinical professor permanent, or fixed at least for a series of years, in the same individuals; and, lastly, by instituting a practical clinic, upon the principle of the poly-clinics of Germany." 19.

We think there cannot be a doubt, among reflecting men, that the only plan of securing the utility of the profession to the public, and the respectability of its members, is to raise the amount, and improve the mode of medical education to the highest degree, compatible with a proper supply for the demands of society. But we are not so Utopian as to imagine that this can be done by levelling all ranks to one denomination, and enforcing one measure of education for all members of the profession. The various gradations in society, and the different modes of remuneration, forbid this equalization and amalgamation. But this we do think, that the present scale of education should be raised in every class of the profession—that of the general practitioner, the operating surgeon, and the physician. Each of these classes is now overstocked, and the inevitable consequence is DISTRESS. It is an old proverb, that "where *Poverty* comes in at the door, *Love* creeps out at the window." This may, unfortunately, apply to the medical profession. The inordinate supply creates a tremendous conflict, not merely for reputation, but for bread! The consequences of this competition need not be descanted on. Now, by enhancing the amount of medical education, we place a check upon the redundancy of medical population, while we ensure to the public the very best assistance in time of need. True it is—"and pity 'tis 'twas true," that this extension of education, and the expences inseparable from it, must prevent the ingress of many a potent mind into the ranks, from sheer want of means to go through the necessary forms. But provision cannot be made for such exceptions. In our, as in other professions, genius and talent must be left to their fate. From time to time they will rise triumphant, in spite of every obstacle opposed by the *res angusta domi*—and what is far worse—by the injustice, the jealousy, and the selfishness of mankind!

We will venture to make one more remark, though it is directly in opposition to the current of popular opinion at the present time. It is this:—That the true and sole test of medical knowledge will not be found in an oral *examination*, however carefully conducted. Yet the universal cry is—let a man acquire his knowledge how, where, and when he pleases—but let him prove his acquisition by the test of examination. This is very fine in theory, but will not work in practice. It is said that, "non ex aliquovis ligno fit mercurius." Be this as it may, we venture to affirm that, by the aid of a good grinder, either in Edinburgh or London, "ex aliquovis ligno fiat medicus." By medicus, we mean a member of any of the three classes of medical society. We shall adduce some facts in illustration. About 30 years ago, when the public services were in great want of medical officers, a considerable number of young men passed examinations under various constituted authorities, (and many of them passed with éclât) without ever having attended a single lecture or dissection, and without any hospital attendance. The proofs of such attendances were not then narrowly scrutinized. We knew a young man, who, having served three or four years with a country apothecary, came to London, and was employed in the shop of an extensive druggist in the city. He plied his books so well, in the evenings and mornings, that he at length ventured to present himself before the Court of Examiners, in Surgeon's Hall. He answered questions with such accuracy and precision, that the Court called him in again, and paid him a public compliment on the extent of his anatomical and surgical acquirements. Yet he had never had one particle of medical education beyond what his master's shop and library afforded him. He had an excellent appointment, in consequence of his brilliant examination, but was soon afterwards drowned in the Bay of Naples. The writer of this article was at Surgeon's Hall at the time this happened, and he knows of *many instances* where young men passed excellent examinations with little or no medical education. Several of these have since turned out to be the most able and useful men which the public services can boast of.

We shall not say any thing of what annually takes place in Edinburgh and London, under the magic influence of the professed grinder. But to shew how imperfectly a man's attainments can be scrutinized by a verbal examination, we must take leave to refer to the recent rejection of Dr. John Mason Good, after an examination by the president and censors of the College of Physicians—all of whom, we believe, were friendly towards the candidate. Here, then, was one of the most erudite physicians of the age in which we live, after being in extensive practice for many years, and after having published numerous medical works of great merit, unable to make his acquirements patent in an examination which a tyro in the profession might undergo—and ignominiously rejected! Again:—One of the most eloquent medical teachers, popular writers, and successful practitioners of the present day, was rejected, a few years ago, in a public examination. Will it be affirmed, with these and many other similar facts before our eyes, that the public examination is the proper test. We say it forms but a part of the test. The most authentic proofs should be required of *a long and regular medical education*, we care not where obtained. This is the *sine qua non;* for the verbal examination is fallacious. There are men capable of being crammed so brim full of technical *learning*, (it is not knowledge) that they will answer all questions in anatomy, pathology, and therapeutics, like parrots, and yet they might scarcely be able to tell one tissue or muscle from another;—or distinguish, at the bed-side, between the most opposite diseases.

In respect to the examination of graduates at Edinburgh, we hold the construction and defence of the thesis as a mere bauble. Every candidate for a diploma should be made to walk through the wards of the infirmary, and shew his clinical acquirements in the investigation of each complaint that presented itself—in remarks upon the nature and pathology of the disease—and, finally, in prescribing for the actual condition and stage of the malady then obtaining. Such a process as this would, at once, separate the grinder's chaff from the wheat of clinical observation, assisted by regular study. If, on this point, the writer of this article should be considered as in any respect visionary—"it can only be, (as Dr. Clark himself has observed) in his believing that what is *done* in other countries, *may be done* in this, and done with a like benefit to the profession of physic."

---

### 9. ACUTE DUODENITIS—ENCEPHALITIS.

The following case is related by Dr. C. F. Bellingheri, of Turin. A female, of robust constitution, and previously enjoying good health, began, towards the end of July, to feel loss of appetite, and depression of spirits. On the 25th August she experienced aversion towards food, and inclination to vomit—tongue foul—pain in the head—no fever. *26th.* Took two ounces of manna, which she vomited. The symptoms continued, and a physician prescribed an emetic of ipecacuan and tartarised antimony. Vomiting followed, but nothing particular was discharged from the stomach—the bowels were then acted on. In the evening of this day Dr. B. saw the patient, and found her with fever; loaded tongue, the point of this organ being red; thirst great; inclination to vomit; no pain, even on pressure, in any part of the abdomen. At this period there prevailed an inflammatory fever in Florence, accompanied by considerable determination to the mucous membrane of the bowels; and, as it was suspected that this patient had made rather too free with strong liquors, Dr. B. did not hesitate to consider the disease as partaking of the reigning epidemic, and as consisting chiefly of inflammation of the stomach and bowels, which would soon affect the head. A copious bleeding was prescribed. The blood was not inflamed. Tamarind water, with sugar, for drink. *27th.* The head-ache had disappeared—thirst continues, vomiting is still distressing—tongue very foul and yellow—sense of taste much depraved—great aversion to food—the food, when swallowed, produced no pain, or heat, or weight in the stomach, nor any increase of the sickness—neither was there any tenderness, on pressure, in any part of the abdomen. The motions were liquid and yellow—urine red, with a sediment. The fever continues, the pulse being quick and concentrated. Another venesection, to the extent of 16 ounces—blood inflamed. Vegetable acid drinks continued. *28th.* Same symptoms: numerous leeches to the anus, followed by syncope. *29th.* The thirst urgent—tongue thickly coated, red at the tip and sides—frequent efforts to vomit—no pain in the abdomen—stools abundant, liquid, and yellow—less fever to day. Ten grains of ipecacuanha exhibited, which was soon returned, merely with the fluids drunk. Things continued in this state, till the 1st September, when the vomiting was almost incessant, and the fever became more intense than before. In the night delirium came on, but she did not complain of pain in the head—cough and crude mucous expectoration. 2d. September, the venesection repeated to 16 ounces—blood buffed, and very consistent. 3d. The delirium has ceased—no pain in the head—none in the abdomen, even on pressure. In the evening, an exacerbation of the fever, and the bleeding was repeated. The blood was now natural, but thick. 4th. The vomiting less frequent—the tongue begins to clean—dejections liquid and black—some miliary eruptions on the skin—general perspiration—urine clear and abundant—pulse rapid, but non-resisting—the patient could give no account of her feelings. These symptoms continued till the 7th, when the head evidently was much affected, and numerous leeches were applied there, while blood was taken from the jugular vein. Blisters, sinapisms, &c. were employed. 8th. Bled from the feet, and ice applied to the head. 9th. Comatose—paralysis of the left arm, with convulsive contractions of the right. There was now pain, on pressure in the region of the duodenum, as evinced by signs, rather than words. 10th. Apoplexy closed the scene.

*On Dissection,* the duodenum was found highly inflamed throughout its whole extent, being of a deep brown colour, and adherent, by means of coagulable lymph, to the neighbouring parts. The stomach was also inflamed, in the vicinity of the pyloric orifice. The rest of the intestinal tube was sound, as were the liver, and other abdominal viscera. In the head, the pia mater was found to be inflamed and injected, particularly over the right hemisphere. The cortical substance of the brain was of a deep ash-colour, and softened. There were more than two ounces of water in the lateral ventricles, of a sero-sanguineous character. "The dissection," says Dr. B. "proves that the disease was a severe acute duodenitis, followed by encephalitis and acute hydrocephalus."

The conclusion which Dr. Bellingheri draws appears to be legitimate, and the case is curious, as shewing that so sensitive an organ as the duodenum may be inflamed, and no pain even on pressure, may be complained of. The emetics in this case, very probably aggravated the disease. The extension of the inflammation, from the intestinal canal to the brain, is here finely illustrated.—*Journal Universel des Sciences Medicales.*

---

### 10. HYPERTROPHY OF THE HEART AS A CAUSE OF APOPLEXY.*

The coincidence which has been for some years past, observed between hypertrophy of the left ventricle of the heart and apoplexy, is now fixing the attention of pathologists. It is reasonable to suppose, on mechanical principles at least, that if the aortic ventricle acquires a preternatural power of propelling the blood towards the brain, in common with other parts of the body, the delicate structure of the encephalic mass may, in time, suffer, and hæmorrhage may be the result. So, again, on the same principle, any obstruction to the free return of blood by the veins, from the head, in consequence of disease about the right side of the heart, should tend to the same disease as violent impulsion from the aortic system. But mechanical doctrines in pathology are always to be taken with some degree of caution, if not distrust. We must bear in mind that, in the great majority of cases of apoplexy, there is no disease of the heart; but a disease in the brain itself or its vessels. There can be little doubt, however, that where this idiopathic affection of the brain exists, the hypertrophy of the left ventricle may prove a powerful accelerator of the apoplectic attack. Be this as it may, we shall report some of the cases brought forward by Dr. Sablairolles, as facts which are independent of doctrines, and from which the reader may draw his own conclusions. This is the safest plan of proceeding, on the part of the journalist, and the most beneficial for the public.

*Case* 1. Jean B. aged 45 years, of small stature, and apoplectic constitution, had long been addicted to immoderate indulgence in wine. Formerly of very mild disposition and temper, he had lately become extremely irascible, and could not bear the least contradiction, without flying

---

* Nouvelles Observations pour servir à l'Histoire de l'Hypersarcose du Ventriell Gauche du Cœur, considerée par Rapport à la Production de l'Apoplexie. Par M. Sablairolles, M.D. de Montpellier.—*Journal Generale de Medecine, Nov.* 1826.

into a violent passion. He was subject to considerable nasal hæmorrhages, which always relieved intense head-aches, vertigo, palpitations, &c. He had had several acute inflammations. At nine o'clock, in the evening of the 26th January 1826, after eating and drinking pretty freely, he became suddenly senseless, and, on recovering a little, he was perceived to be unable to articulate his words. He was carried to bed, and our author found him with the following symptoms:—Coma, face of a deep red colour, lips blue, mouth drawn to one side (left,) pulse hard, contracted, and irregular, action of the heart tumultuous, great difficulty of breathing, deglutition impossible, general insensibility, involuntary discharge of urine and fæces. Cold was applied to the head, sinapisms to the legs and feet, leeches to the temples, lavements, &c. In three quarters of an hour—or in two hours from the accession of the attack, he breathed his last.

*Dissection.* The membranes of the brain were extremely red, and the whole cerebral vascular system greatly distended with blood. On slicing the brain, the blood flowed at each cut of the scalpel. The most rigorous examination could not detect any trace of extravasation of blood in the head.

The heart was more than double its natural size. The parietes of the left ventricle were greatly thickened, and the cavity diminished. The origin of the aorta was enlarged. Nothing particular observable in the right chambers of the heart, except that they were gorged with blood. The mucous membrane of the stomach and small intestines was inflamed.

This is one of the cases which have been occasionally recorded by Morgagni and others since his time, where apoplexy quickly destroyed life, without any escape of the blood from its proper channels, but only from the general distention of those channels, by which the brain was universally compressed. These are the kinds of apoplexy which most rapidly destroy life, or else quickly disappear. If the universal compression is not relieved, the functions dependent on the brain must soon cease; for it is not as in cases of extravasation, where one half or more of the brain remains uninjured, and carries on the functions though imperfectly. On the other hand, these apoplexies from congestion are sometimes more speedily and more easily removed than those dependent on hæmorrhage. There is very little evidence, in the above case, that the apoplexy was induced through the instrumentality of the hypertrophia ventriculi sinistri; for, if the parietes of the ventricle were thickened, the capacity of the chamber was lessened—so that although the momentum of the blood might be augmented, the quantum sent from the heart, at each systole, was diminished.

*Case* 2. Badollin, aged 69 years, had long been a prey to domestic chagrin and misfortunes, at the commencement of which, (18 years previously,) he had an attack of difficulty of breathing, which lasted six weeks, and was attended with violent palpitation of the heart. Since that period, these attacks have occasionally returned, but at long intervals, and not so severe in degree as at first. Towards the close of 1818, these paroxysms assumed all their pristine violence. The action of the heart was precipitate and tumultuous—the recumbent posture on the left side was impossible, and the difficulty of breathing was great. To these symptoms was added a severe pain in the right side of the chest. An eruption took place on the skin of the left side of the thorax, and quickly the symptoms above-mentioned were mitigated. The patient went on rather better till September, 1819, when he was obliged to give up his occupations. The palpitation and dyspnœa were now in a most violent degree—his legs began to swell—effusion took place in the abdomen—the intellectual faculties became disturbed—the œdema reached the upper extremities, and in this state he was received into the Clinique Interne de la Faculté, where he died in a few days.

*Dissection.* Besides the general œdema of the extremities, there was sanguineous infiltration into the substance of the brain, and water in the ventricles. There was a large collection of yellow serum in the right side of the chest, and the lung of that side almost entirely hepatised. There was no water in the pericardium;—the heart was covered with much fat, and was nearly double its natural size. The chambers were dilated, and the parietes of the left ventricle were "visibly thickened." There was a red tinge (which could not be washed off) in the lining membrane of the left ventricle, and on the internal surface of most of the large vessels. The liver was enlarged, and there were unequivocal marks of phlogosis in the mucous membrane of the stomach.

*Remarks.* We do not see any cogent reason for supposing that the sanguineous infiltration of the brain, in this case, was solely owing to the hypertrophy of the heart. Doubtless the cardiac disease, and the hepatised state of the right lung, were the causes of the general serous effusions; and it is probable that what was called *sanguineous* infiltration in the cerebral substance, was merely *sero-sanguineous* effusion, and not hæmorrhage. The symptoms of apoplexy are no where stated as having occurred before the death of the patient.

*Case* 3. M. G. aged 54 years, had enjoyed good health till June 1821, when he had an attack of hepatitis occasioned by distress of mind. This gave way to proper treatment, and two months afterwards he was seized with an intermittent, (quartan) which resisted every kind of treatment till the end of December of the same year. In the beginning of January 1822, M. G. on getting out of bed, became suddenly deprived of sense, and on reviving a little, was found hemiplegiac and unable to speak. He was bled largely and repeatedly, and in two days speech and muscular power were restored. He complained of pain in the head, vertigo, and tinnitus aurium. Leeches, pediluvia, antimonials, &c. apparently restored the patient, in a few days, to health. More than two years elapsed, after this, without any accident, except that he had occasionally frightful dreams, and his breathing was short, on going up an ascent. He was now destined to experience a fresh attack, which ended in paralysis of the right side. This hemiplegia had continued four months, when our author was consulted. The patient complained of violent palpitation, difficulty of breathing, acute head-aches, vertigo, and an irresistible propensity to sleep after meals. The face was injected, the eyes prominent, pulse strong, quick, and irregular. After examining the heart attentively by auscultation, our author diagnosticated an hypertrophy of the left ventricle. Bleeding, general and local, rigid abstinence, digitalis, counter-irritants were prescribed. At this interview the sudden decease of a near relation by apoplexy, was imprudently announced to the patient, the effect of which was, *instantaneous death by the same disease!*

*Dissection.* The membranes of the brain were very much injected, and of a red colour. The right hemisphere presented no appearance of disease. In the centre of the anterior lobe of the *left* hemisphere was a small cavern, the size of a pigeon's egg, filled with black and coagulated blood. The cerebral substance surrounding this apoplectic cyst was changed into a kind of bouillie of a yellowish colour. In the same hemisphere was found, near the fissura magna sylvii, a large quantity of blood, apparently of recent extravasation, and which had partly made its way into the lateral ventricle of that side. This extravasation was evidently the immediate cause of death. There was no alteration of the cerebral structure in the vicinity of this effusion.

The lungs were sound—the heart very much enlarged—the parietes of the left ventricle more than an inch and a half in thickness—auricles dilated, and their coats very thin—nothing remarkable in the abdominal viscera.

This case exemplifies the fact, that a large effusion of blood in the brain, as well as universal congestion, may occasion sudden death. The cyst in the left hemisphere of the brain corresponded with the previous hemiplegia of the right side.

One of the three cases above detailed shews that we are not always to expect a corresponding cyst for each preceding attack of apoplexy. If there be an extravasation of blood, and a succeeding paralysis of any duration, we may be pretty certain of finding the said cysts. Where the apoplectic attack is not followed by distinct paralysis, we may generally conclude, that the pathology of the disease was congestion merely, without rupture of vessels.

How far the foregoing cases corroborate the opinion of hypertrophy of the heart being a cause of apoplexy, we leave to the judgment of our readers. For ourselves, we have no doubt of the truth of this etiological doctrine generally speaking; but we think that one or two of the cases, here detailed, are of equivocal validity on this point, though interesting in other respects.

---

### 11. REGENERATION OF THE URETHRA.

In our last Number, page 220, we gave the particulars of a case of "regeneration of sphacelated urethra," by Dr. Brown of Sunderland, as published in the Ed. Journ. Med. Science, No. 4. We request that our readers will turn to that case, and re-peruse it, as it is very short. It will be seen, that we doubted not the veracity of Dr. Brown's statements, but the conclusions which he drew from what appeared to him as facts. We have received a long letter from Dr. B. vindicating himself from the imputation of being deceived, or of wishing to deceive others. The *latter* was never imputed to him, and therefore we shall pass it by unnoticed. That he was deceived, is no great imputation; for we believe that there is not a medical man in existence, who has been ten years in extensive practice, and who has not been *a thousand times* deceived. Dr. B. hopes we will "cast off, or rather cast out, the slough of scepticism," in our next number. Dr. B. who is a man of great literary acquirements, and deep philosophy, ought to know that it is not in a man's power to alter either his creed or his scepticism by a voluntary effort. Both are the result of conviction; and there is nothing in Dr. Brown's letter which has shaken our scepticism. It is true Dr. B. has taken the advice and opinion of several eminent practitioners on the case, and they all give it in his favour. This must be very gratifying to him; and we can assure him it is not in the least mortifying to us. The case is fairly before the public, and let that public decide.

One fact, however, which Dr. B. has now brought forward, in proof of the correctness of his opinion, that it was the urethra itself which was extruded from the wound in the left side of the abdomen, deserves to be recorded. It is this:—that the man has since lost all venereal *appetite*. He accounts for this phenomenon, by supposing that the apertures seen in the preparation are the openings of the prostatic ducts. Now, without referring to musty volumes on this subject, we shall appeal to the experience and observations of surgeons. When the prostatic ducts are cut, or obliterated by inflammation, after lithotomy, the accident *does not destroy*, or even impair, the venereal appetite. It prevents the emission of the seminal fluid, and occasions great distress, of which we have recently seen a remarkable instance, but we maintain that it does not destroy the sexual appetite. These facts before our eyes, no statements in books can make us "cast off the slough of scepticism." Dr. B. no doubt considers us as quite solitary in our scepticism. We beg him, therefore, to peruse the Revue Medicale for November last, pages 280—3, where he will find that the "slough of scepticism" will require his caustic on both sides of the channel. The French reviewer observes, that "tout doit etre *merveilleux* dans cette observation;" and concludes with the following sentence: "Toutefois, nous devons dire ici que l'auteur n'a pas donné assez de details, ni sur la maladie, ni sur la piéce pathologique, *pour lever tous les doutes* que se fait laissera dans l'esprit des lecteurs."

It is but fair to state, that Dr. Brown means to send the preparaticn to London, in which case we promise to submit it to two or three of the first anatomists of this capital, and publish the result of their examination. Unquestionably those who have seen the preparation have better means of forming a judgment, than we who have only seen the plate. Dr. B. will perceive, that the French journalist is not convinced, any more than ourselves, by the drawing, and therefore we recommend him to send the original to town.

### 12. REMARKABLE DISEASE OF THE STOMACH.

The following extraordinary case is related by M. Nacquart, secretary-general to the Medical Society of Paris, in the January number of the Journal General de Medicine.

*Case.* Madame S. aged 50 years, had been subject to derangement of the digestive functions all her life, accompanied sometimes by pains in the epigastrium, at others, by nausea or vomitings. These symptoms were mitigated by warm baths, abstemiousness, and amusements, and she had occasionally long intervals of tolerable health. In 1822, the catamenia ceased, and then there came out an herpetic, furfuraceous, and even erysipelatous eruption on the face, which continued more than three months. Some time after this, Madame S. remarked, that the left hypochondrium was somewhat enlarged, as well as all that side of the abdomen. She also felt darting or dragging pains in her stomach. The intumescence was attributed to enlargement of the spleen; meanwhile the epigastrium became tender on pressure, and the stomach refused to digest the lightest aliment. In 1825, she had occasional vomitings of black matters, like coffee grounds, with copious ptyalism of mucous matters from the mouth. The tongue was denuded and painful—constipation obstinate. On the 27th March, 1826, M. Nacquart was called to the patient, and found her excessively emaciated, with an indolent tumour communicating the sensation of a gellatinous fluid, occupying the left hypochondriac fossa, stretching over to the epigastrium, and downwards below the umbilicus. Pressure on this tumour caused borborygmi, and percussion elicited a hollow sound. There was nothing particular in the constitutional symptoms. By a strict regimen, the use of a bandage, and taking the waters of Vichy, the patient mended greatly, the gastric irritability subsiding, and the tumour decreasing in size. But the amendment was of short duration, and the vomitings returned, with general malaise, and sense of burning pain in the stomach. These symptoms again subsided—again the appetite returned—and what remained of the tumour was soft, but still sonorous. The case was so mysterious, that Dupuytren was called in consultation (5th of June), but he could not decide on the nature of the tumour. He was of opinion, however, that the tympanitic character must be owing to some convolutions of intestine placed over it. During the next month, the appetite continued pretty good, but at the expiration of that period, the black vomitings again returned, preceded, as before, by ptyalism, and thus blasting the hopes which the patient had entertained of a recovery. The tumour, in the centre of the epigastrium, now became more apparent, more tender, and the emaciation increased, with œdema of the lower extremities, and diarrhœa. *27th July.* The tumour, for some days past, had become so enlarged, as to dip into the left side of the pelvis. At this time the patient discharged from her stomach more than three pints of a black fluid, mixed with the debris of food formerly taken. Her condition was now very distressing, and she could keep no aliment on her stomach. On the 7th of August, while sitting down in her chair, she uttered a cry, bent herself forwards, and with a faint voice averred, that something had given way internally, and produced a burning sensation in the abdomen. She died in 12 hours from this period.

*Dissection.* The peritoneum was inflamed in several places, and contained two or three pints of turbid fluid, mixed with flocculi and pus. The abdomen being completely opened, the stomach appeared to be divided into two portions—the one on the left forming an immense pouch, descending into the hollow of the ilium, and concealing the whole mass of small intestines on that side—the other portion, or pyloric, not larger in volume than the duodenum. These two portions were connected by a contracted portion, in front of the vertebral column, and in this portion was a circular perforation, about four lines in diameter. The stomach having been entirely isolated, the left division was found to contain about two pints of a brownish fetid matter, mixed with streaks of pus, its mucous lining being softened, discoloured, and presenting extensive arborisations. The peritoneal coat was pale and healthy—the right, or pyloric portion, three or four inches in length, was about the size of the duodenum, and might have been mistaken for it, if the pylorus had not remained intact. The mucous membrane of this division was somewhat thickened, but otherwise sound. The middle, or intervening portion, presented an irregular mass, three or four inches in length, and of unequal diameter, varying from one to two inches. Its parietes were indurated, and there was a canal through its centre of only some lines in calibre. When this portion was slit open, it shewed an irregular ulcer, of cancerous aspect, with ragged edges, and with several softened tubercles in its substance, some containing encephaloid matters, some a black and diffluent substance. In one of the sinuosities of this ulceration there was a circular perforation, as above-mentioned. The matters contained in the abdomen resembled those found in the stomach. Some convolutions of the intestines were phlogosed and adherent, but the mucous membrane of the duodenum, and all the intestines, were perfectly healthy. The liver and other organs were sound.

It would be useless to speculate on the origin and progress of this terrible disease. The case shews the difficulty of exactly ascertaining the nature of abdominal tumours.

### 13. CEPHALO-SPINAL FLUID.

Two things often puzzled us when examining the bodies of the dead—the disproportion of size between the spinal marrow, and the canal in which it is contained—and the fact that, in almost all cases, there was more or less serous effusion (as it is called) at the base of the skull, and in the vertebral canal. In a late sitting of the Royal Institute of Paris, M. Magendie read a memoir on the fluid found in the cranial and spinal cavities of man and mammiferous animals, to which fluid he has given the epithet "Cephalo-rachidien." This eminent physiologist commences with an estimate of the total amount of this fluid, which he found to vary, in the adult and healthy subject, from two to five ounces. Among other uses, M. Magendie considers the cephalo-spinal fluid to serve the purpose of keeping, in a proper degree of plenitude, the cavities of the cranium and spine. In old age, the shrinking up of the brain and spinal marrow (not sufficiently observed by physiologists) causes a greater space for the accumulation of this fluid, and is thus inimical to the sustenance of life. In corroboration of this opinion, M. Magendie has observed, that, in the old and emaciated females who die at the Salpetriere, the quantity of this cephalo-spinal fluid is very considerable. Numerous experiments on animals have demonstrated, that this same liquid, when drained off artificially, is very quickly reproduced, like the humours of the eye. The effect which this artificial evacuation produces in animals, is a state of numbness and hebetude, which continues till the reproduction of the liquor. In two instances, he found the evacuation induce a state of agitation and violence in the animals, which resembled hydrophobia, and lasted for three or four days. An artificial augmentation of the cephalo-rachidien liquor causes pressure on the brain and spinal marrow, and determines paralysis. The disease, known by the name of spina bifida, consists, according to M. Magendie, of a kind of hernia of the membranes which contain the said fluid. By pressure of the spina bifida, in children, he has produced the same phenomena which were exhibited by animals, where injections had been used to augment the quantum of this fluid. The temperature of the cerebro-spinal liquid was found to be under that of the blood, by some degrees. M. Magendie has produced in animals a tremor and a momentary paralysis, by drawing off this liquor, suffering it to cool, and then injecting again the same fluid. These symptoms appeared to last till the fluid had regained its natural temperature. If, after the evacuation of the fluid, the same was injected again, of the natural temperature, no such symptoms occurred. The motion of the head on the trunk of the animal, produced a wave or agitation of the fluid in the spine. M. Magendie is of opinion, that the contact of this fluid plays an important part in the development of electricity, and has undertaken a series of experiments, with the view of determining this point. All physiologists now agree in believing, that a certain quantity of halitus, or watery vapour in the ventricles of the brain, is consistent with health. M. Magendie has proved, to his own satisfaction at least, that a communication exists naturally between the lateral and the other two ventricles of the brain, and between the fourth ventricle and sub-arachnoid cavity of the spine. This communication, he says, is established by an aperture between the two posterior cerebellic arteries, and is, at least, three lines in diameter. M. Magendie proposes to call this aperture "entreé des cavitiés du cerveau," or "entrée des ventricules cerebraux."

The communication being thus established between the ventricles of the brain and the spinal canal, M. Magendie avers that, in all morbific affections of the brain, as acute and chronic hydrocephalus, in which there is an undue dilatation of one or more ventricles, these "entrées," or communications, become very much enlarged. The experiment of pressure on a spina bifida affecting the head proves the communication in the living body. The following experiment was made by M. Magendie :—he injected four ounces of ink, very gently, into the lower portion of the vertebral canal. This was sufficient to blacken, not only the whole surface of the brain, but the internal surfaces of all the ventricles. Whenever pressure was made on the spinal envelopes, a fresh quantity was pushed into the third ventricle.

In more than fifty bodies of people who had died without any cerebral affection, M. Magendie found from half an ounce to two ounces of fluid in the ventricles of the brain. Care should be taken, however, that the body be not placed in a position to favour the draining of the ventricular fluid into the spinal canal. This eminent physiologist is of opinion that, if the cephalo-rachidien liquor amount to more than two ounces, certain morbid phenomena will be the result, especially serous apoplexy. M. Magendie terminates his memoir with these words :—"is it not remarkable that those parts of the brain, called by the old anatomists, *valvule, aqueduct, bridge,* should have precisely the uses which their names import? It is thus that the valve of Vieussens, or valvula magna cerebelli, performs the office of a valve (soupage) and opposes the exit of fluid from the fourth ventricle. Never did a part better merit its name than the *aqueduct of Sylvius,* since, according to the experiments which I have made, this canal transports the fluid, sometimes from the ventricles to the spine—sometimes from the spine to the head. Finally, that part which has been termed a *pons,* is, in fact, an arcade of medullary matter, placed over the current of fluid which traverses the aqueduct."—*Revue Medicale, Janvier.*

### 14. ACUTE RHEUMATISM.

For several years past, we have occasionally taken opportunities, as they presented themselves, to protest against the indiscriminate practice of blood-letting in acute rheumatism. Venesection is usually carried to a very great extent in this disease ; and the state of the blood, the hardness of the pulse, the local inflammation, and other phenomena, appear to justify the depletory measures, in their fullest extent. Yet the course of the disease is seldom shortened by free depletion, even in strong constitutions ; while, in weak habits, or where there is a tendency to any disease of an internal organ, we not unfrequently find some metastatic action set up, not, indeed, at the very time of the rheumatic attack, but at a longer or shorter period afterwards, which is of much more serious consequence than the original malady. For many years past we have been in the habit of making minute enquiries into the previous histories of those who have laboured under organic disease of the heart, especially dilatation of that organ, with or without thickening of the parietes. In by far the greater number of cases, rheumatism, of the acute or subacute kind, had preceded the cardiac affection ; and the majority of these asserted that they had been freely bled for the rheumatic complaint. It is true that this species of evidence, as to cause and effect, is far from being demonstrative ; but, unfortunately, it is such as we are too often obliged to put up with in medical enquiries.

In the Medical and Physical Journal, for February, Dr. A. T. Thomson has detailed four cases of rheumatism, and made some observations on the disease, which we shall briefly notice. Dr. T. conceives that modern experience has "thrown doubts upon the *inflammatory nature* of rheumatism, and altered the treatment of the disease." He instances the striking resemblance between acute rheumatism and ague, as noticed by Hulse, Morton, and others. "The pain," says he, "when th[illegible]isease is left to run its course undisturbed, *recurs* periodically every night o[illegible]ery morning, once, at least, in 24 hours." The term "*recur*," however, is only applicable to certain forms of rheumatism; for, in the common acute rheumatism, of every day occurrence, we have remissions and exasperations of the pain, but rarely, if ever, *intermissions*. Where we have regular periodical *recurrence* of the pain, it is then intermittent rheumatic *irritation*, rather than inflammation. In common acute rheumatism, where we have all the attributes of inflammation, dolor, rubor, calor, tumor, we do not see how the "inflammatory nature" of the disease can be doubted. But it does not follow that every inflammation is to be cured by bleeding. Nobody doubts that, when the foot, in gout, is hot, painful, red, and swelled, there is inflammation; but experience has proved that this phlogosis cannot be removed, at least with safety, like that which follows a local injury in a healthy constitution, by bleeding, &c. The specific nature of an inflammation must always modify the treatment. This is the case with acute rheumatism. It is a specific inflammation; often taking on the form of periodical or intermittent irritation.

Dr. Thomson believes that the dread of metastasis, in this disease, has led to the discontinuance of bark; "but experience has demonstrated that this dread is unfounded, and where the employment of this remedy has been prefaced by the exhibition of calomel, tartar-emetic, and opium, with cathartics, he has had no reason whatever, in a practice of twenty-five years, to alter his opinion of its efficacy and safety."

*Case* 1. A young lady, after exposure to a current of moist air, felt a sudden pain at the inner angle of the left orbit, which was slightly swelled. It extended thence along the brow, and disappeared in the evening. Next day it returned, and disappeared as before; and this went on for eight days, when Dr. T. was called in. The accessions were characterized by febrile and inflammatory symptoms; but the remission every evening was complete, and the lady slept well. Dr. T. prescribed calomel and hyosciamus at night, with saline aperients in the morning, in which was a drachm of the vinum colchici. The next paroxysm was later, but equally severe. Dr. T. prescribed one grain of calomel, a grain and a half of opium, and a quarter of a grain of tartar emetic, every six hours, *dolore urgente*; and a mixture of oil of turpentine with the cinchona, every four hours, *absente dolore*. The paroxysms not only returned later, but became much milder, and soon ceased altogether.

It is curious, and, perhaps, fortunate, that the same disease will often be cured by a great variety of remedies. The above is a very common case in practice. In many instances, we stopped the disease by bark and arsenic, exhibited during a single interval. Such decidedly intermittent irritations or inflammations, as the above case, scarcely require any preparation for that specific, which almost inevitably stops their progress.

*Case* 2. This patient was also a young lady, who, after a slight shiver in the evening, was seized with such violent pain over the whole head, that Dr. T. was roused out of bed at midnight to attend her. There was no heat of skin —feet cold as marble—feels as if her head were screwed in a vice, and it is sore to the touch—pulse is small and low, &c. Magnesia, colchicum, æther, and camphor julep—pediluvia—castor oil in the morning. There was a remission only of the pain next day. The bark was given till the evening exacerbation came on. It was violent as before. Next morning, a more marked remission. The bark continued, and an emetic to be taken on the accession of the evening paroxysm. *Third day.* There was but very little pain after the operation of the emetic. It returned, however, at midnight. The bark continued—a dose of blue-pill, antimony, and opium, at bed-time. The paroxysms now became later and milder, and soon disappeared.

We cannot think that Dr. Thomson means to designate the above as a case of "acute rheumatism," though it is so headed. There was no pyrexia throughout the complaint. The general state of the pulse was "small and sixty"—the skin cool, or even cold. This is not *acute* rheumatism, but a periodical rheumatic irritation.

The third case related by Dr. Thomson is of a similar nature to the two preceding. The fourth case is the only one which has any pretension to the designation of "acute rheumatism." The patient was a servant, who had been exposed to wet and cold, and was then seized with severe pain in the wrists and ancles. In two days it shifted to the loins, and was accompanied by some heat of skin and fever, with the usual perspirations. He had a daily rigor, succeded by febrile re-action. Calomel, followed by saline aperients, with colchicum, were given—calomel and opium at night—and oil of turpentine, with bark, through the day. By these remedies he was soon cured.

These cases are selected by Dr. Thomson from among many others, chiefly on account of the combinations in which the cinchona was administered. "The oil of turpentine, in particular, has always appeared to increase the efficacy of the bark." In some constitutions it affected the kidneys, but not often. We have no doubt that Dr. Thomson's plan is a very good one—and, as we said before, several other plans may be equally successful. In the common acute rheumatism we seldom bleed, unless the fever runs very high, and the patient is plethoric. Even then we bleed sparingly. Some calomel and anodyne at night, with saline aperients and colchicum through the day, generally bring the disease to a remittent or even intermittent form, when we give bark and arsenic to finish the cure.

---

15. PHLEBITIS—CONSECUTIVE ŒDEMA. PHLEGMASIA DOLENS.

We are of opinion that the paper of Dr. Davis on PHLEGMASIA DOLENS, published in the 12th volume of the Medico-Chirurgical Transactions, has given, and continues to give, an erroneous view of the pathology of the above-mentioned disease, both in this country and on the continent. The experience of medical practitioners has determined phlegmasia dolens to be a disease so rarely fatal, that it was very difficult to find on record a dissection of a case; but now, by identifying the said disease with inflammation of the pelvic or crural veins, we have satis superque of pathological investigations. It was only this night (27th Feb. 1827) that a paper was read, and a preparation sent round, in the Medico-Chirurgical Society, shewing a case of phlegmasia dolens in the male, with the crural vein inflamed and plugged up—adding one more proof that the cause of the disease is phlebitis. The more experienced and observant members of the society, however, refused to admit that the case was one of phlegmasia dolens at all—but one of phlebitis, a very different disease, whether we contemplate the symptoms or the termination. There is no doubt, indeed, from the cases published in this and other countries, that *inflammation and obstruction of the iliac veins* may take place, and produce the swelling of the limb, and the death of the patient. But if this were the pathology of phlegmasia dolens, we should have as few instances of recovery as we have now of death, in that disease. Besides, in all the cases which have been brought forward for the support of this doctrine, the swelling was quite of a different nature from that of phlegmasia dolens. It was *œdematous*, while the swelling in the genuine disease is *elastic*. Lastly, we may add that, in the case published by Dr. Denmark, of phlegmasia dolens, in a male, and which proved fatal at Haslar Hospital, the vein was *not* inflamed or plugged up. In the discussion which took place at the Medico-Chirurgical Society, a case of genuine phlegmasia dolens, in the puerperal state, was brought forward by Dr. Granville, in which the new pathology failed, as there was no phlebitis. Several members, and among others, Dr. Tweedie, physician to the fever-hospital, shewed that the disease which is now confounded with genuine phlegmatia dolens, is often seen to take place after puerperal peritonitis, and even common fever; but is quite different in character from the genuine disease.

In respect to the case which was read by Dr. Forbes, the swelling of the leg and thigh took place in the last stage of consumption, and the limb was œdematous in every part. The iliac and inguinal veins were inflamed, and obstructed by a concrete and fibrinous plug. The young man died of the pulmonary disease.

We shall now proceed to some cases brought forward by M. Bally, of Paris, for the support of the new doctrine, of which he is a stanch advocate.

*Case* 1. This was the case of Caroline Dunn, detailed by Dr. Davis, and of which we took ample notice in the first volume of this series, page 378-90.

*Case* 2. This case is recorded by M. Melier. The patient was a female 30 years of age, who, having come to the full term of her second pregnancy, was taken in labour. The child lay crosswise, and the accoucheur proceeded to turn; but while in this operation, the patient screamed violently, and he was forced to desist. A second and a third attempt was also unsuccessful. The woman, however, becoming more weak, and more placable, the child was ultimately turned, and the delivery effected. For the first 36 hours after her accouchment, nothing particular occurred; but at this period, she complained of acute pain in the right iliac region, accompanied by general malaise, and rigors. On the third day, she had fever—the abdomen was tender and tense, especially on the right side. The milk subsided this day from the breasts, and the symptoms indicated approaching peritonitis. Venesection—leeches to the abdomen—fomentations. In spite of these means, the peritonitis made rapid progress. At this time (28th July, 1826,) Dr. Melier was called in, and learnt the preceding history. The pain was now very acute in the belly, particularly in the right side, where the slightest pressure, the succussion of a cough, or any movement of the body, occasioned insupportable torture. Obscure fluctuation was detected in the right ileo-lumbar region. The fever was considerable—the lochia suppressed—pulse small and frequent. The *patient was bled copiously by the application of* 40 *leeches*. Warm bath—fomentations—lavements. 29th, The symptoms were still more severe to-day, and 30 more leeches were applied. Dry cupping was frequently practised on the breasts this day, by which means considerable swelling and some lacteal secretion were induced. There appeared to be some mitigation of the abdominal inflammation. 30th, Rather better. 31st, An exasperation of the symptoms—more leeches—blisters, &c. 1st August, sensibly better to-day. The pain in the right side of the abdomen is not so severe; but a fluctuation there is still perceptible. From this time there was a gradual amendment, the pain diminishing, and the tumefaction of the abdomen subsiding. Hopes were entertained of her recovery, and Dr. Melier discontinued his visits. The accoucheur continued in attendance; although the fever did not entirely cease, it was very much reduced. There came on, in a few days, a slight discharge from the vagina, of a sanguinolent character, and very fetid; but it was considered to be the remains of the lochia. In a few days more, the discharge suddenly became abundant, and of a purulent appearance. This continued day and night, and was at any time, increased by pressure on the abdomen. The matter was, at first, inodorous, but became afterwards greenish and fetid—the fever assumed the hectic form—debility made great progress. The pain in the ileo-lumbar region was now renewed, and presently the lower extremity became infiltrated, first about the foot, then the leg, and ultimately the thigh. On the 16th August, Dr. Melier again came in attendance, and found the patient in a desperate condition, the right leg and thigh swelled and painful. She died on the 17th of the same month.

*Dissection.* The peritoneum presented universal traces of inflammation, more intense in the right than in the left side. In the right iliac fossa there was a vast abscess, with numerous surrounding adhesions of the intestines. This abscess communicated with the vagina. The uterus was healthy. The external iliac vein, and three or four inches of the crural vein were completely obliterated by a clot of blood and fibrin, resembling a polypous concretion. The parietes of these veins were thickened, and the internal membrane red. There was some pus in the veins. All the other vessels of that side were unaffected.

Here then is one of the cases which are now assimilated with phlegmasia dolens, and from which the pathology of the disease is attempted to be established. We should consider it an insult to the practical reader, if we were to stop a moment to shew how dissimilar are the two diseases. Who is it that will not, at once, perceive that the iliac vein, in the above case, partook of the destructive inflammation going on within the abdomen of that side, and thereby became obstructed. That the swelling of the limb was, in a great measure, occasioned by this obstruction to the return of blood, we allow; but all swellings are not of the same nature, nor do they all arise from the same cause; and again we aver that this disease was not phlegmasia dolens.

*Case* 3. Observed by M. Guiette, surgeon to St. Peter's Hospital, Brussels. The patient was an unmarried female servant, aged 22 years, and who had never been pregnant. She was admitted into the hospital, in the year 1824; for the treatment of a considerable swelling of the lower left extremity—" resembling the œdema which is often seen in puerperal women."* In all other respects, the patient's health was good—all the functions natural and regular. The most accurate examination could not discover any cause for this swelling. No treatment was adopted. In the course of a few days, however, the swelling of the limb became much increased, the pain more intense, and the tension excessive. To these were added constitutional symptoms of irritation, and disorder of the digestive organs. Her nights were now passed in dreadful sufferings. The surgeon in chief

* This is the general expression; but whenever the swelling comes to be particularized by the abettors of the new doctrine, no resemblance whatever is found between the two diseases.—*Ed.*

determined to make scarifications on the inside of the thigh, from which *an abundance of serum issued.* By this remedy and proper regimen, together with fomentations, &c. the malady seemed to yield, and in six weeks the limb was reduced to its normal dimensions. She was discharged apparently cured. But in the course of the next year, the patient returned, with the limb still more swelled than before; and now the swelling had attacked both the lower extremities. The least movement excited insupportable sufferings—the tumefaction was at its utmost degree—*and the swelling pitted on pressure of the finger.* The health of the patient was greatly deranged—digestion difficult—stomach unable to bear, without pain, the lightest nourishment—bowels constipated—breathing short, difficult, and laborious—sanguification imperfect, and face of a blueish tint, as in hydrothorax—pulse small, quick, and irregular—urine obliged to be drawn off daily. At this time, there was felt in the hypogastric region, a hard globular mass, which was supposed to be a scirrhous ovary, or enlarged uterus.

The disease made rapid progress—emaciation advanced—eschars formed on the back, and the patient died.

*Dissection.* There was no disease in the chest. The abdominal viscera presented nothing extraordinary. On the front of the lumbar vertebræ was a large tumour of a fatty lardaceous nature, in the midst of which was found the ascending cava, greatly enlarged, its diameter being more than two inches and a half. On prosecuting the dissection, the primitive iliac and hypogastric veins, their principal divisions, the cava ascendens, as far as the diaphragm, were found extremely dilated, and filled with a dense, whitish, fibrinous-looking substance, in the centre of which there was a small canal, one line in diameter, through which the venous blood returned from the lower parts of the body. The parietes of these vessels were somewhat thicker than natural, and they were surrounded by degenerated cellular substance. The aorta was not affected. The lower extremities were infiltrated, and their veins much dilated.—*Journal Gen. de Med. January,* 1827.

Here is another case of *phlegmasia dolens !*—another proof that the cause of the disease is inflammation and obstruction of the pelvic or iliac veins! It is only necessary to clearly and fairly state these cases, to convince every observant and experienced practitioner that they are not the disease known by the term (however improper) *phlegmasia dolens.*

But the discussion of the new doctrine is very advantageous, and all these cases are interesting specimens of pathology, which are highly deserving of record.

Were we pressed for our own opinion as to the nature of phlegmasia dolens, we would say that it approaches nearer to what is termed diffuse cellular inflammation than to any other malady with which we are acquainted—but that it is not identical with that. In the diffuse cellular inflammation there is a "boggy" feel, to use the expression of Dr. Duncan and others; while in phlegmasia dolens, it is a tense elastic feel. This last disease, too, is not near so dangerous as the former. It is, therefore, a peculiar, or specific malady, *sui generis,* the proximate cause of which is not yet ascertained. But, from all that we have yet seen or heard, we are satisfied that this proximate cause is *not* PHLEBITIS.

### 16. HERNIA OF THE STOMACH THROUGH THE DIAPHRAGM.

At a late meeting of the Westminster Medical Society, a remarkable case of wound of the diaphragm was detailed by Mr. Hunt. The particulars are as follow:—The patient was stabbed in the left side with a knife, and for a month afterwards his life was in jeopardy. He then recovered from the effects of the wound, but from this time began to suffer from dyspepsia. When Mr. Hunt first saw the patient, nearly 16 months after the injury, he had cough, but unaccompanied by pain, or inability to expand the chest, attended by a peculiar kind of vomiting, apparently more dependent on the action of the stomach itself, than on that of the abdominal muscles and diaphragm. There was no febrile action, the pulse being below 70. Twelve ounces of blood were taken from the arm. Next day, the symptoms being much exasperated, a larger quantity of blood was abstracted, and with relief to the patient. To check the slow inflammation which appeared to be going on in the stomach, blisters, &c. were applied to the epigastrium. For eight days the case seemed doing well, but at that time a low remittent fever was set up, which, however, at the end of a week or so, had nearly subsided under proper treatment. In the course of a day or two he became anxious, and was attacked with retching and vomiting of matters resembling coffee-grounds. From this period no food could be retained upon the stomach, nor could any passage be procured through the bowels, and on the 5th day he died exhausted.

*Dissection.* On raising the sternum, a large mass was seen, which at first was thought to be an enlarged heart and pericardium, but which turned out to be the stomach greatly distended, and situated entirely in the left cavity of the thorax. On exposing the cavity of the abdomen, there was found a large opening in the diaphragm to the left, and rather below that for the passage of the œsophagus; it had a smooth and rounded margin, and through it the stomach and part of the duodenum had passed into the chest. It was with great difficulty, owing to the distension of the viscus with fluid, that it could be drawn back through this opening into the abdomen. The lung was apparently uninjured, and the intestinal canal sound, but near the pylorus there were marks of chronic inflammation, with ulceration of the mucous membrane.

*Remarks.* This is certainly a curious case. The manner in which the stomach got into the thorax, seems, in spite of a good deal of hot discussion which took place on it, simple enough. The knife pierced the diaphragm most probably in expiration. Through this wound a portion of the stomach was forced, partly, no doubt, by the suction power of the chest, as Dr. Barry well observed, but principally by the pressure of the abdominal muscles, and descent of the diaphragm itself. The opening continued to enlarge, and more of the stomach protruded through it, giving rise to irritation, and chronic inflammation of its coats, as shewn by the dyspepsia and vomiting. At last the whole of the organ, dragging with it part of the duodenum, became located in the thorax, and it was at this time, in all probability, that the violent retching and vomiting occurred, and that the passage of food through the alimentary canal ceased. The preparation is sent to the museum of the College of Physicians.

The above are the remarks of our correspondent; but we have some doubt as to the supposed wound of the diaphragm in this case. It is not impossible that there might have been a congenital opening in the diaphragm, although the hernia might not have occurred till after the accident above detailed. The fallacy of the " post hoc, ergo propter hoc," proposition is well known. The Editor of this Journal examined the infant of a medical gentleman (Dr. Golding), in which there was a large congenital opening in the diaphragm, through which the stomach accidentally passed, and caused the death of the child. Another instance, still more remarkable, has lately been communicated by M. Sigaud to the Royal Society of Medicine of Marseilles, of which we shall here state the particulars.

A young man, 21 years of age, died suddenly, after a fit of indigestion, and the body was examined for the purpose of ascertaining the cause of this sudden and mysterious death. The dissection was performed by M. Sigaud, in the presence of Messrs. Giaud and Magail, when the following notes were recorded. In the left side of the diaphragm there existed a circular aperture, with rounded edges, and at least three inches in diameter, through which aperture a large mass of intestines had penetrated into the left cavity of the thorax, and had there become strangulated, causing the death of the patient. The preparation is preserved in the museum of the Society.—*See Archives Generales. Janvier* 1827, *p.* 130.

Now, had this young man received a penetrating wound in the direction of the diaphragm previously to this sudden death, it might, and probably would, have been said, that the aperture was thus produced, and that death had taken place in consequence of the opening left in the muscular partition between the two cavities.

---

### 16. REMITTENT BRONCHO-ENCEPHALITIS.

Under this head M. Broussais has related a curious case in his Journal (*Annales de la Med. Physiolog.*) for October last. The patient, a young man, was attacked with symptoms indicative of inflammatory action in the bronchial membrane, and also in the brain, with violent exacerbations, during which the cough was incessant, and the cephalalgia greatly aggravated by the successions of the cough. After three bleedings from the arm, and several applications of leeches to the temples and neck, the disease took on a decidedly periodical character, and then the sulphate of quinine was administered, with an allowance of light nourishment, especially as no symptoms of gastric irritation were present. The appetite was good, and the tongue clean. The medicine but slightly mitigated the paroxysms of coughing, and presently the skin became permanently hot and dry, the bowels irritable, and the embarrassment of the chest increased, the expectoration being viscid, and brought up with difficulty. The sulphate of quinine was discontinued, and leeches were applied to the lower part of the trachea. The strict antiphlogistic regimen was again adopted. This plan was crowned with success. " What," says M. Broussais, " would have been the result, if we had persevered with the febrifuge, because the disease assumed a periodical character? The patient having lost much blood, the inflammation might not, perhaps, have assumed an acute character, but certainly a chronic gastro-enteritis would have been established, and possibly the bronchial inflammation would have been converted into suppurative pneumonia."

